# Laccase Gene Expression and Vinasse Biodegradation by *Trametes hirsuta* Strain Bm-2

**DOI:** 10.3390/molecules200815147

**Published:** 2015-08-19

**Authors:** Raúl Tapia-Tussell, Daisy Pérez-Brito, Claudia Torres-Calzada, Alberto Cortés-Velázquez, Liliana Alzate-Gaviria, Rubí Chablé-Villacís, Sara Solís-Pereira

**Affiliations:** 1 Laboratorio GeMBio, Centro de Investigación Científica de Yucatán A.C., Calle 43 No. 130, Chuburná de Hidalgo, Mérida 97200, Yucatán, Mexico; E-Mails: daisypb@cicy.mx (D.P.-B.); ctorres@cicy.mx (C.T.-C.); betocv@cicy.mx (A.C.-V.); rubi.chable@cicy.mx (R.C.-V.); 2 Unidad de Energía Renovable, Centro de Investigación Científica de Yucatán A.C., Calle 43 No. 130, Chuburná de Hidalgo, Mérida 97200, Yucatán, Mexico; E-Mail: lag@cicy.mx; 3 Departamento de Ingeniería Química y Bioquímica, Instituto Tecnológico de Mérida, Av. Tecnológico Km 5, Mérida 97118, Yucatán, Mexico; E-Mail: ssolis@itmerida.mx

**Keywords:** *Trametes hirsuta*, lacasses, gene expression, biodegradation, vinasse, phenolic compounds

## Abstract

Vinasse is the dark-colored wastewater that is generated by bioethanol distilleries from feedstock molasses. The vinasse that is generated from molasses contains high amounts of pollutants, including phenolic compounds and melanoindin. The goal of this work was to study the expression of laccase genes in the *Trametes hirsuta* strain Bm-2, isolated in Yucatan, Mexico, in the presence of phenolic compounds, as well as its effectiveness in removing colorants from vinasse. In the presence of all phenolic compounds tested (guaiacol, ferulic acid, and vanillic acid), increased levels of laccase-encoding mRNA were observed. Transcript levels in the presence of guaiacol were 40 times higher than those in the control. The *lcc1* and *lcc2* genes of *T. hirsuta* were differentially expressed; guaiacol and vanillin induced the expression of both genes, whereas ferulic acid only induced the expression of *lcc2*. The discoloration of vinasse was concomitant with the increase in laccase activity. The highest value of enzyme activity (2543.7 U/mL) was obtained in 10% (*v*/*v*) vinasse, which corresponded to a 69.2% increase in discoloration. This study demonstrates the potential of the Bm-2 strain of *T. hirsuta* for the biodegradation of vinasse.

## 1. Introduction

Vinasse (molasses distillery wastewater) is residual wastewater that is derived from the production of bioethanol. It is dark brown in color and is rich in organic matter and toxic compounds, such as phenolic compounds and melanoidins [1,2]. For each liter of bioethanol obtained, 10 L of vinasse are produced. The disposal of this residue is hazardous and presents a considerable risk of pollution that is increased when this wastewater is used in agriculture. In the Mexican state of Veracruz, the high volume of bioethanol production by the alcohol and sugarcane industries amplifies this risk. Thus, research into alternative biotechnological processes for the effective degradation of phenolic compounds and the discoloration of vinasse is of the utmost importance [3,4].

White-rot basidiomycetous fungi are notable for their ability to degrade a diverse range of environmentally persistent xenobiotics and organopollutants [5].

A complex non-specific ligninolytic enzymatic system secreted by these fungi is thought to be involved in the degradation of a wide variety of natural and synthetic materials, such as lignin, melanoidins, tannins, humic acids, polycyclic aromatic hydrocarbons, and chlorophenols. The most studied enzymes that are capable of breaking down colored pollutants in wastewater include manganese peroxidase, lignin peroxidases, and laccases, which are capable of breaking a large quantity of different chemical bonds [3,6]. Laccases are produced in the presence of several inducers, and their effects on metabolic activity and cell growth are dependent on environmental conditions and specific regulatory mechanisms [7,8]. Laccase catalyses the reduction of O_2_ to H_2_O using a range of phenolic compounds as hydrogen donors [9]. They can detoxify phenols via oxidative coupling and polymerization and reduce the phenol content of different wastewater [10].

The goal of this work was to study the expression of laccase genes in the *Trametes hirsuta* strain Bm-2, isolated in Yucatan, Mexico, in presence of phenolic compounds, and its effectiveness in the removal of colorants from vinasse.

## 2. Results and Discussion

### 2.1. Effects of Aromatic Compounds on the Expression of Laccase Genes

As shown in Table 1, all of the aromatic compounds used in this study had an inductive effect on the level of laccase activity, with guaiacol showing the highest induction of enzymatic activity.

**Figure 1:**
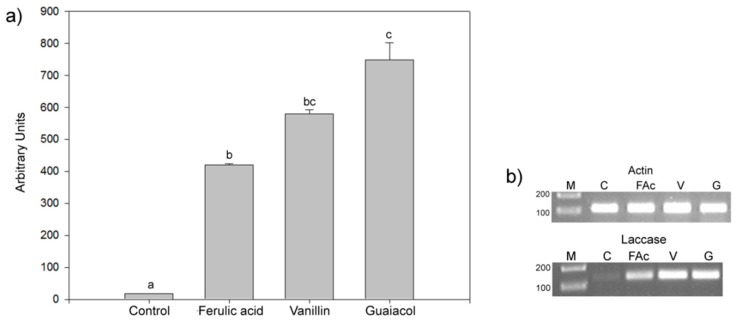
(**a**) The effects of ferulic acid (FAc), vanillin (V), and guaiacol (G) on laccase activity; (**b**) *lcc* ARNm gene transcript levels in eight-day-old cultures of *T. hirsuta* Bm-2. A fragment of the actin gene was used as an internal control for each sample. C: control (cultures in Kirk’s medium without the addition of any aromatic compound). Results were represented as means ± standard deviation of three parallel measurements (*n* = 3). Different letters represent significant differences at *p* < 0.05.

An analysis of the inductive effects of phenols on the transcription of *T. hirsuta* Bm-2 laccase genes is presented in Figure 1. Whereas a considerable increase in laccase gene transcript was observed in the presence of all phenolic compounds compared with the control (Figure 1a), the largest increase was obtained with guaiacol (40.4 times), followed by vanillin (33.3 times) and ferulic acid (24.15 times). A quantitative analysis was performed by calculating arbitrary units of expression, which showed that the total transcript level of laccase mRNA following the addition of guaiacol was higher than those obtained with the other two phenols, thereby corroborating the laccase activity data presented in Table 1. Figure 1b shows the PCR products obtained for actin and laccase. The actin PCR products were of equal intensity across all samples, excluding the possibility that the apparent laccase level was affected by inhibitors of cDNA synthesis while normalizing the gene expression level. Guaiacol produced the best overall induction of *lcc* gene transcription.

**Table 1:** Production of *T. hirsuta* (Bm-2) laccase in basal medium supplemented with different aromatic compounds.

Compound	Chemical Structure	Laccase Activity (U/mL)
Ferulic acid	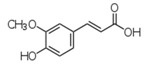	380
Vanillin	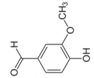	450
Guaiacol	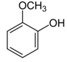	1700
Control (Kirk’s medium)	---	150

Aromatic compounds, such as phenols, are structurally related to lignin and play an important role in the level of laccase production by basidiomycetes. One of the main factors determining the effectiveness of induction is the chemical structure [11].

Increased induction may be the result of either transcription factors directly increasing the mRNA level or a post-transcriptional modification, such as an increase in the stability of the lacasse mRNA transcript [12,13].

Guaiacol, which induced the highest levels of laccase mRNA, is a compound that has been used in many studies because of its ability to induce laccase activity in *Trametes* sp. in different effluents [5]. Ferulic acid and vanillin are structurally similar to guaiacol, and although they positively influence laccase gene transcription, they have been reported to induce lower levels of laccase, in agreement with the results obtained herein [11].

**Figure 2:**
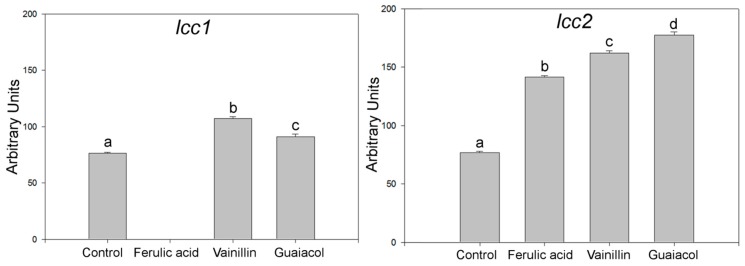
*lcc1* and *lcc2* transcript levels as determined by the densitometric quantitation of RT-PCR products. The relative rates of *lcc1* and *lcc2* transcription following the addition of different phenolic compounds to eight-day-old *T. hirsuta* Bm-2 cultures are presented in arbitrary units (AU). Results were represented as means ± standard deviation of three parallel measurements (*n* = 3). Different letters represent significant differences at *p* < 0.05.

In the quantitative analysis of *lcc1* and *lcc2* mRNA (Figure 2), we observed differences in the response to each phenolic compound. For *lcc1*, the highest value (111 AU) was obtained in response to vanillin; however, for *lcc2*, the highest induction was produced with guaiacol (185 AU), followed by vanillin and ferulic acid (169 and 144 AU, respectively).

These results indicate that *T. hirsuta* Bm-2 laccase genes (*lcc1* and *lcc2*) are specifically regulated by phenolic compounds and that, in general, the compound with the highest influence on enzyme production was guaiacol. Similar results were observed for the laccase genes *lcc1*, *lcc2*, and *lcc3* in the *Trametes* sp. strain CECT 20197 [11,12].

### 2.2. Fungal-Treated Vinasse

**Figure 3:**
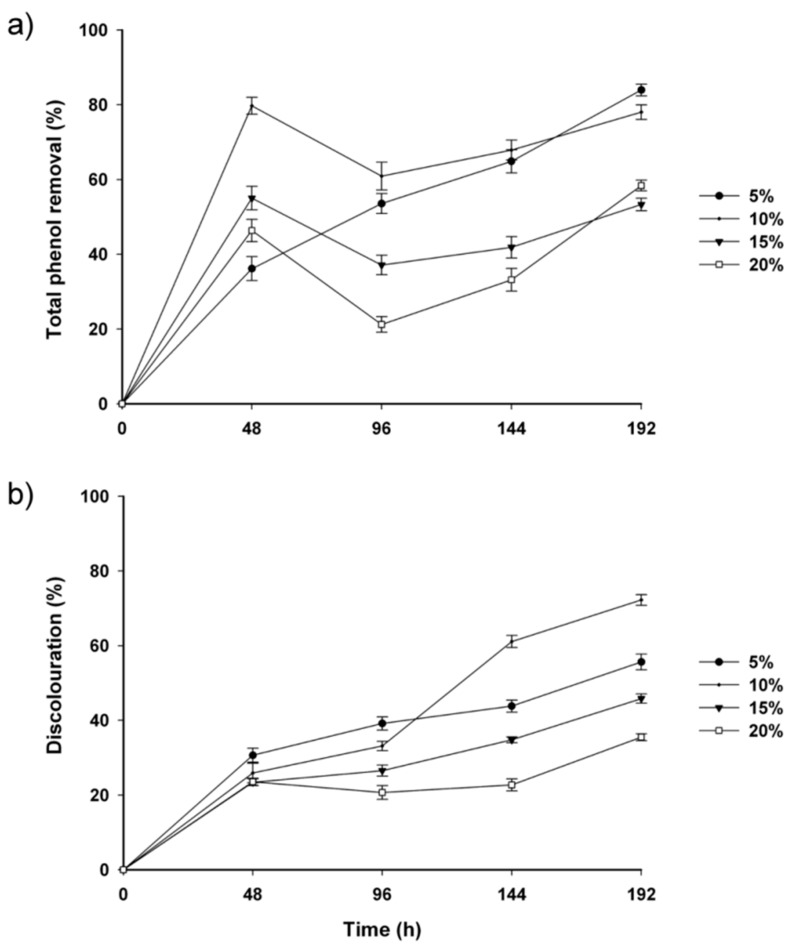
(**a**) Total phenol removal and (**b**) percentage of vinasse discoloration by *T.* *hirsuta* Bm-2 in different vinasse concentrations over 192 h of cultivation. Results were represented as means ± standard deviation of three parallel measurements (*n* = 3).

The fungus *T. hirsuta* Bm-2 exhibited an increase in phenolic compound removal and discoloration after 48 h in different concentrations of vinasse (Figure 3). There was a considerable difference in the total phenolic compound removal between 5% and 10% (*v*/*v*) and 15% and 20% treatments (*v*/*v*). The greatest phenol removal (Figure 3a) was observed in 5% and 10% (*v*/*v*) vinasse at 192 h (84.8% and 79.2%, respectively), meanwhile, at the same time, 15% and 20% (*v*/*v*) treatments showed relatively low total phenolic compound removal effects (53.3% and 58.8%, respectively).

Color elimination ranged between 35% and 72% (Figure 3b); the highest discoloration (72.23%) was achieved in 10% (*v*/*v*) vinasse at 192 h, followed in decreasing order by 5%, 15%, and 20% (*v*/*v*) treatments with 55.6%, 45.8%, and 35.5% discoloration, respectively. Vinasse is characterized by the presence of high molecular weight polymers called melanoidins, which are formed by phenolic compounds undergoing the Maillard reaction [2]. These substances are frequently toxic to the microorganisms used for the biotreatment of effluents, being highly recalcitrant and possessing antioxidant properties [3,14,15,16].

Several studies of the degradation of melanoidins and related compounds have been performed using white-rot fungi (e.g., *Phanerochaete chrysosporium*, *Ganoderma* sp., and *Trametes* sp. I-62). This discoloration was associated with the activity of ligninolytic enzymes (laccase and manganese-peroxidase) [2,3,5]. Many reports have been published claiming that polyphenoloxidases have notable detoxifying effects [17].

The presence of phenolic compounds and color removal could be attributed to fungal degradation rather than simple physical binding; similar results were achieved using *Trametes* sp. for the biotreatment of distillery wastewater [5]. The results obtained in the present study demonstrate the potential of *T. hirsuta* Bm-2 as an organic compound-degrading agent.

**Figure 4:**
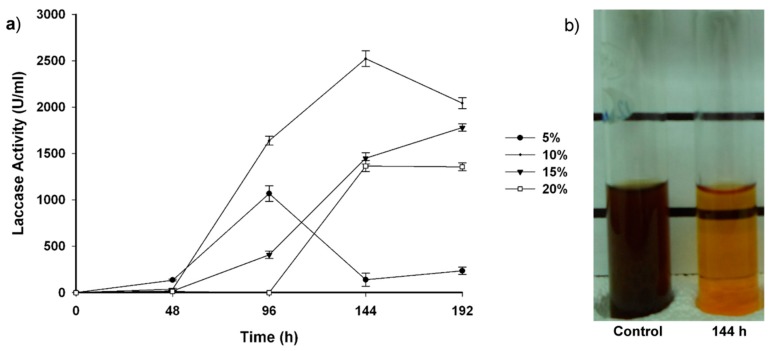
(**a**) Laccase activity of *T. hirsuta* Bm-2 over eight days of cultivation in different vinasse concentrations; (**b**) Discoloration of 10% (*v*/*v*) vinasse by *T. hirsuta* Bm-2 at 144 h of cultivation. Results were represented as means ± standard deviation of three parallel measurements (*n* = 3).

In this study, the cultivation of *T. hirsuta* Bm-2 in 10%, 15%, and 20% (*v*/*v*) vinasse resulted in high laccase activity over an eight-day incubation period (Figure 4a). Extracellular laccase production increased significantly in 10% (*v*/*v*) vinasse (2543.7 U/mL) at 144 h of cultivation.

The highest increase in laccase activity observed under the conditions assayed in our study corresponded to a high vinasse discoloration value (Figure 4b), suggesting that laccase overproduction may play an important role in vinasse discoloration and that phenolic compounds, such as ferulic acid, vanillin, and guaiacol, induce the production of transcripts encoding this isoenzyme. The low laccase activity (234.7 U/mL) in 5% (*v*/*v*) vinasse might be a consequence of the low phenolic compound concentration (as laccase-mediator) in the medium. Meanwhile the laccase activity values, 1777. 7 U/mL (15%) and 1355.5 U/mL (20%) vinasse, might be due to a negative effect of high concentrations of phenolic compounds and melanoidins on the expression of these enzymes. Some of the phenolic compounds found in various wines and in molasses distillery wastewater have been reported to negatively affect microbial growth [2,18,19]. Phenolics can also inhibit the aerobic degradation of molasses distillery wastewater [20].

Similarly, a significant increase in laccase activity is frequently observed in our laboratory when *T. hirsuta* Bm-2 is grown on other industrial effluents, such as textile dyes [21]. Similar results were obtained when *Trametes* sp. I-62 was used for the biotreatment of distillery wastewater [5].

Together, our results suggest the potential of *T. hirsuta* Bm-2 for use in the removal of organic compounds and pigments present in vinasse. Considering the large volume of molasses distillery wastewater generated in the production of bioethanol, this fungus could play an important role in the treatment of vinasse such that it may be used in fertigation.

## 3. Experimental Section

### 3.1. Fungal Strain, Media, and Culture Conditions

*Trametes hirsuta* strain Bm-2 was isolated from decaying wood in Yucatan, Mexico [21], and used throughout this work. Cultures of this fungus were maintained on Kirk’s liquid basal medium, pH 6, which consists of 10 g glucose, 5 g ammonium tartrate, 0.2 g MgSO_4_·7H_2_O, 2 g KH_2_PO_4_, 0.1 g CaCl_2_·2H_2_O, 1 mg Thiamine, and 10 mL trace compound solution, without Tween 20 or veratryl alcohol [22,23]. To determine the effect of aromatic compounds (ferulic acid, vanillin, and guaiacol) on *lcc* gene transcription, the compounds were added to four-day-old Bm-2 strain cultures (35 °C, 150 rpm) at a final concentration of 0.5 mM. Control samples were prepared under the same conditions without the addition of any aromatic compound. Fresh mycelium samples (approximately 10 mg) were harvested at 72 h after the addition of the compounds to the cultures [24].

### 3.2. Total RNA Preparation and cDNA Synthesis

RNA extraction was performed using the RNAspin Mini RNA Isolation kit (GE Healthcare, Buckinghamshire, UK) following the manufacturer’s instructions.

First-strand cDNA synthesis was performed using 200 ng of total RNA as a template and 500 μg/mL of primer dT_12–18_, with the enzyme SuperScript II RT from Life Technologies (New York, NY, USA) according to the manufacturer’s instructions.

### 3.3. PCR Amplification

cDNA corresponding to transcripts from the Bm-2 strain was amplified with different primer combinations: Primer I (F): 5′-TGG CAY CAY TTY GGN TTY CA-3′ and Primer II (R): 5′-RTG RCT RTG RTA CCA RAA NGT-3′, which flank the sites linked to copper I and II [25], and *lcc*1 (dil15 5′-atg gga agc tgc agt tc-3′/dil13 5′-gca cga gag cga aac taa-3′) and *lcc*2 (dil25 5′-atg tcg agg ttc cac tc-3′/dil23 5′-aac gcg gac cac gct tcg c-3′) primer pairs [26].

PCR amplifications were performed in a 25 μL reaction mixture containing 1X PCR buffer (20 mM Tris-HCl, 50 mM KCl, pH 8.4; Invitrogen, Carlsbad, CA, USA), 0.3 mM concentrations of each deoxy-ribonucleoside triphosphate (dNTP, Invitrogen), 2.5 mM MgCl_2_, 1 μM concentrations of each primer, 2 μL of template cDNA, and 1.25 U of *Taq* polymerase (Invitrogen). The reaction was performed on a GeneAmp 9700 DNA Thermal Cycler (PerkinElmer, Life Technology, Carlsbad, CA, USA) according to a previously reported protocol [26].

PCR products were visualized on 1.5% (*w*/*v*) agarose gels prepared in 1X Tris-Borate-EDTA (Invitrogen) buffer and stained with ethidium bromide. Densitometry analysis was performed with a UVP BioImaging System (UVP. Inc., Upland, CA, USA) using Lab Works 4.0 software.

The following primers were used to amplify the housekeeping gene actin: Act-512F 5′-ATGTGCAAGGCCGGTTTCGC-3′ and Act-783R 5′-TACGAGTCCTTCTGGCCCAT-3′ [27]. Amplifications were performed in the thermal cycler GeneAmp 9700 DNA Thermal Cycler (PerkinElmer), which was programmed as follows: an initial denaturation of 5 min at 94 °C; 30 cycles consisting of: denaturation, 30 s at 94 °C, annealing, 30 s at 61 °C, and extension, 90 s at 72 °C; and a final extension of 7 min at 72 °C. Each reaction was performed in 25 μL of reaction mixture containing 1X PCR buffer (20-mM Tris-HCl, 50-mM KCl, pH 8.4; Invitrogen), 0.2-mM concentrations of each dNTP (Invitrogen), 2.5-mM MgCl_2_, 1.5-μM concentrations of each primer, 2 μL of template cDNA, and 1.5 U of *Taq* polymerase (Invitrogen).

### 3.4. Quantitative and Statistical Analyses

PCR products (10 μL of each reaction) were separated by 1.5% agarose gel electrophoresis and visualized after staining for 10 min in a 1 μg/mL ethidium bromide solution. Densitometry analysis of gel images was performed using UVP BioImaging Systems software (Lab Works 4.0; UVP Inc, Upland, CA, USA). Levels of *lcc* mRNA were expressed in arbitrary units, and the relative rate of appearance for the *lcc* transcript level (previously normalized based on size differences) and actin was calculated by the following equation: laccase/[actin_(sample)_/actin_(average)_]. For all experiments and determinations, the variability coefficients between the three replicate samples were calculated. Significant differences were determined by the t-test for mean comparison (with *p* < 0.001).

### 3.5. Vinasse

Vinasse samples were obtained from Ingenio La Gloria (Veracruz, Mexico), an industrial distillery that uses molasses as a raw material for ethanol production and that generates vinasse as wastewater. Samples were taken from a container while still hot (70 °C) and were bottled for subsequent cooling and refrigeration (4 °C).

### 3.6. Preparation of Vinasse-Containing Medium

First, 250-mL Erlenmeyer flasks were filled with 100 mL of vinasse diluted by the addition of distilled water to obtain concentrations of 5%, 10%, 15%, and 20% (*v*/*v*) in solution. The pH of these solutions was adjusted to 4.5. Then, 2 mL of fungal inoculum was inoculated into each flask and subsequently incubated for 196 h with orbital rotation (130 rpm) in a New Brunswick shaker at 28 ± 2 °C. Samples were collected every 48 h. Three replicates were incubated for each concentration.

### 3.7. Total Phenolic Content and Discoloration

The concentration of total phenolics was determined by the Folin-Ciocalteu method [28], which measures the formation of a blue complex spectrophotometrically at 740 nm following the reduction of a phosphomolybdic-phosphotungstic reagent by phenolics. The phenolic content of the samples was expressed as gallic acid equivalents. The discoloration was calculated according to the formula utilized by Sirianuntapiboon *et al.* [29] according to the absorbance measurements at 475 nm. The reported color removal was calculated according to the following equation:

(1) $$\text{Discoloration (\%) = }\frac{(\text{ODi – ODf)}}{\text{ODi}}\text{ × 100}$$

where ODi = initial absorbance and ODf = final absorbance.

### 3.8. Laccase Activity

The liquid cultures obtained after fungal biomass separations were spectrophotometrically analyzed for enzyme activity at 25 °C. Laccase activity was determined using the substrate 2,2′-azino-bis (3-ethylbenzothiazoline-6-sulfonic acid) (ABTS) and was measured at 420 nm (ε_max_ = 36,000 L·mol^−1^·cm^−1^) [30]. One unit of laccase activity was defined as the amount necessary to catalyze the formation of 1 mmol of oxidized ABTS min^−l^.

### 3.9. Statistical Analysis

Analyses of all experiments were carried out using Analysis of Variance (ANOVA) with 95% confidence limits. Values were averaged and given with the standard deviation (± SD) as shown in Figure 3 and Figure 4. The statistical significance of the results was tested at *p* < 0.05 level using Tukey’s test. All statistical analyses were performed using SAS 9.1 (SAS Institute, SAS Campus Drive, Cary, NC, USA).

## 4. Conclusions

In our study, all phenolic compounds resulted in an increase in laccase activity, with the highest levels detected in the presence of guaiacol. The two laccase isozyme genes expressed by this fungus, *lcc1* and *lcc2*, are differentially expressed in the presence of different aromatic compounds. The discoloration of and phenol compound removal from vinasse were concomitant with an increase in laccase activity, indicating the potential for the Bm-2 strain of *T. hirsuta* in vinasse biodegradation.
